# Increasing Vitamin C Content in Plant Foods to Improve Their Nutritional Value—Successes and Challenges

**DOI:** 10.3390/nu5093424

**Published:** 2013-08-30

**Authors:** Daniel R. Gallie

**Affiliations:** Department of Biochemistry, University of California, Riverside, CA 92521-0129, USA; E-Mail: drgallie@citrus.ucr.edu; Tel.: +1-951-827-7298; Fax: +1-951-827-4434.

**Keywords:** l-ascorbic acid, ascorbate, photosynthesis, DHAR, MDAR, reactive oxygen species

## Abstract

Vitamin C serves as a cofactor in the synthesis of collagen needed to support cardiovascular function, maintenance of cartilage, bones, and teeth, as well as being required in wound healing. Although vitamin C is essential, humans are one of the few mammalian species unable to synthesize the vitamin and must obtain it through dietary sources. Only low levels of the vitamin are required to prevent scurvy but subclinical vitamin C deficiency can cause less obvious symptoms such as cardiovascular impairment. Up to a third of the adult population in the U.S. obtains less than the recommended amount of vitamin C from dietary sources of which plant-based foods constitute the major source. Consequently, strategies to increase vitamin C content in plants have been developed over the last decade and include increasing its synthesis as well as its recycling, *i.e.*, the reduction of the oxidized form of ascorbic acid that is produced in reactions back into its reduced form. Increasing vitamin C levels in plants, however, is not without consequences. This review provides an overview of the approaches used to increase vitamin C content in plants and the successes achieved. Also discussed are some of the potential limitations of increasing vitamin C and how these may be overcome.

## 1. Introduction

In addition to its roles in cardiovascular function, immune cell development, and iron utilization, vitamin C (l-ascorbic acid) serves as a water-soluble antioxidant in animals [1,2,3]. Despite the fact that most mammals can synthesize ascorbic acid (Asc), humans are an exception as a result of a mutation to l-gulono-1,4-lactone oxidase, the last enzyme in the animal Asc biosynthetic pathway [4]. Because Asc is water-soluble, it is not stored and is readily excreted from the body. Therefore, Asc must be obtained regularly from dietary sources. The National Academy of Sciences has recommend 90 mg/day of the vitamin for adult males and 75 mg/day for adult females. Although vitamin C can be obtained from the consumption of fresh meat, it is destroyed by heating and is more typically obtained from plant sources. Asc is present in high amounts generally in fruits and leafy vegetables whereas grains typically have much lower levels of the vitamin, particularly in dried grain. Moreover, the diet of a significant portion of the global population consists largely of plant-based foods. Although post-harvest reductions in Asc can occur, particularly in leafy vegetables, increasing Asc content would help to preserve the nutritional quality of stored foods. As a result, much research has focused on developing strategies to increase vitamin C content in plant foods to improve their nutritional value including strategies to increase the biosynthetic capacity of plants and to increase the recycling of Asc once it has been used in a reaction [5,6,7].

**Figure 1:**
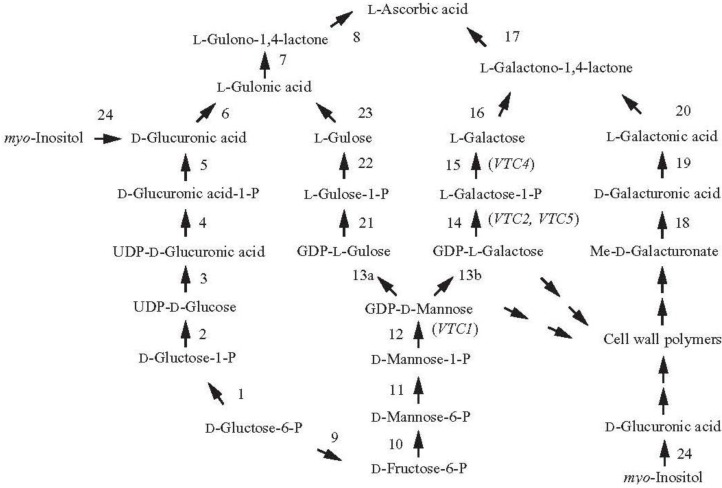
Plants and animals employ distinct pathways for the synthesis of l-ascorbic acid. The pathway in animals is represented by reactions 1-8 whereas the pathways in plants are represented by reactions 9–24. Enzymes catalyzing the reactions are: 1, phosphoglucomutase; 2, UDP-glucose pyrophosphorylase; 3, UDP-glucose dehydrogenase; 4, glucuronate-1-phosphate uridylyltransferase; 5, glucuronate 1-kinase; 6, glucuronate reductase; 7, aldonolactonase (gluconolactonase); 8, gulono-1,4-lactone oxidase or dehydrogenase; 9, glucose-6-phosphate isomerase; 10, mannose-6-phosphate isomerase; 11, phosphomannose mutase; 12, GDP-mannose pyrophosphorylase (mannose-1-phosphate guanylyltransferase) (*VTC1*); 13, GDP-mannose-3′,5′-epimerase; 14, GDP-l-galactose phosphorylase (*VTC2* and *VTC5*); 15, l-galactose-1-phosphate phosphatase (*VTC4*); 16, l-galactose dehydrogenase; 17, l-galactono-1,4-lactone dehydrogenase; 18, methylesterase; 19, d-galacturonate reductase; 20, aldonolactonase; 21, phosphodiesterase; 22, sugar phosphatase; 23, l-gulose dehydrogenase; 24, *myo*-inositol oxygenase.

## 2. Increasing Vitamin C Content through Improved Biosynthesis

The pathway of vitamin C synthesis in mammals begins with d-glucose and proceeds through d-glucose-1-P, UDP-glucose, UDP-d-glucuronic acid, UDP-d-glucuronic acid-1-P, d-glucuronic acid, l-gulonic acid, and finally gulono-1,4-lactone (Figure 1). Gulono-1,4-lactone oxidase then converts gulono-1,4-lactone into 2-keto-gulono-γ-lactone which spontaneously converts to l-ascorbic acid [8].

In contrast to this single pathway, there are at least four biosynthetic pathways suggested to date in plants. The first discovered was the Smirnoff-Wheeler pathway in which Asc synthesis originates with l-galactose [9] (Figure 1). l-Galactose is produced from mannose-1-phosphate through the intermediates guanosine diphosphate (GDP)-mannose and GDP-l-galactose [10]. l-Galactose then undergoes oxidation to l-galactono-1,4-lactone catalyzed by the NAD-dependent l-galactose dehydrogenase followed by oxidation to l-ascorbic acid by the mitochondrial-localized l-galactono-1,4-lactone dehydrogenase [11,12].

Feeding experiments provided support for the Smirnoff-Wheeler pathway. For example, feeding leaf tissue with the Asc precursors l-galactose or l-galactono-1,4-lactone resulted in their conversion to Asc and therefore increased Asc content [9,13,14]. In another study, exogenous application of l-galactono-1,4-lactone to *Arabidopsis* or *Medicago sativa* leaves increased foliar Asc content up to 8-fold and was proportional to the amount applied [15]. Application of l-galactono-1,4-lactone or l-galactose to source potato leaves also increased the Asc content of these leaves as well as in sink organs, e.g., flowers and developing tubers [16].

*Arabidopsis* mutants affected at different steps in the Smirnoff-Wheeler pathway resulted in substantial reductions in Asc content, supporting the conclusion that this pathway is responsible for much of the Asc biosynthetic capacity in this species. For example, the *vtc1* mutant lacks GDP-mannose pyrophosphorylase expression whereas the *vtc2* and *vtc5* mutants lack GDP-l-galactose phosphorylase expression. The *vtc1* mutant exhibits a 70%–75% reduction in Asc content while the *vtc2* mutant contains just 10%–20% of the wild-type level of Asc, *vtc5* contains 80% of the wild-type level, and the *vtc2*/*vtc5* double mutant bleaches in the absence of exogenous Asc or l-galactose which overcomes the block in the pathway [13,17,18]. The *vtc4* mutant results from a mutation in l-galactose-1-P phosphatase [19,20].

Attempts to increase Asc content through increasing its biosynthesis have achieved some success. Overexpression of GDP-l-galactose phosphorylase from kiwifruit (*Actinidia chinensis*) increased Asc content in tobacco leaves by more than 3-fold with an accompanying 50-fold increase in enzyme activity [21]. Although the agroinfection approach employed resulted in only a transient increase in enzyme expression, up to a 4-fold increase in Asc content was observed in stably-transformed *Arabidopsis* where the enzyme was overexpressed [21,22]. Stable transformation of GDP-l-galactose phosphorylase into potato, tomato, and strawberry resulted in up to a 3, 6, and 2-fold increase in Asc, respectively, in tubers and fruits, although some loss of seed and the jelly of locular tissue surrounding the seed were observed in tomato and an increase in polyphenolic content was observed in strawberry and tomato [23]. A combinatorial approach in which kiwifruit GDP-l-galactose phosphorylase and GDP-mannose-3′,5′-epimerase were transiently overexpressed in agroinfected tobacco leaves increased Asc content up to 7-fold [22]. Overexpression of l-galactose dehydrogenase, which catalyzes the conversion of l-galactose to l-galactono-1,4-lactone (Figure 1), however, failed to increase foliar Asc content in tobacco despite a 3.5-fold increase in the activity of the enzyme [24], suggesting that the endogenous level of l-galactose dehydrogenase is not rate-limiting. Transformation of Arabidopsis with GDP-galactose guanylyltransferase resulted in a 2.9-fold increase in Asc and co-transformation with either l-galactose-1-phosphate phosphatase or l-galactono-1,4-lactone dehydrogenase increased Asc content up to 4.1-fold [25]. Overexpressing multiple enzymes within the Smirnoff–Wheeler pathway, particularly those whose endogenous level is closest to being rate-limiting, may offer more promise to achieving substantial increases in Asc rather than the overexpression of any one enzyme. The choice of which enzymes to overexpress may be species-dependent as the level of expression for each enzyme in the pathway may differ among species.

Evidence for other biosynthetic pathways has suggested three alternative routes for the synthesis of Asc. In the first of these alternative pathways, d-galacturonic acid, generated from the breakdown of pectin during fruit ripening, serves as the starting point for Asc synthesis and is reduced to l-galactonic acid as catalyzed by the NADPH-dependent d-galacturonic acid reductase (GalUR) [26] (Figure 1). l-Galactonic acid spontaneously converts to l-galactono-1,4 lactone which l-galactono-1,4-lactone dehydrogenase converts to Asc [26]. Early support for this pathway came from the observation that d-galacturonic acid-1-^14^C was metabolized to l-ascorbic acid-6-^14^C through an inversion pathway in detached ripening strawberry fruit [27]. Supplying a methyl ester of d-galacturonic acid to cress seedlings and *Arabidopsis* cell cultures also increased Asc [28,29], suggesting that GalUR expression was not confined to fruits. Expression of the GalUR gene from strawberry increased whole-plant Asc content 2- to 3-fold in *Arabidopsis* [30], supporting the existence of this pathway. Demonstration that GalUR can increase foliar Asc biosynthesis through d-galactonic acid and d-galacturonic acid suggests that the substrates for this pathway are present in leaves. The potential for manipulating this pathway to achieve increased Asc content will depend on whether the enzymes of the pathway are expressed and whether d-galacturonic acid is present, e.g., following pectin degradation.

An example of the contingent basis of this pathway was observed in developing tomato fruit. Feeding tomato plants with d-galacturonate failed to increase Asc content in immature green tomato fruit while feeding with l-galactose, representing the d-mannose/l-galactose (or Smirnoff–Wheeler) pathway, did increase Asc content [31]. In contrast, feeding of either precursor increased Asc content of red ripened fruits, correlating with the increase in activity of d-galacturonate reductase and aldonolactonase, the last two enzymes of the d-galacturonate pathway in ripe fruits [31]. These observations suggest that the d-galacturonate pathway is not operative prior to ripening during which pectin is degraded. Thus, the contribution that the d-galacturonate pathway makes to Asc biosynthesis in tomato fruit may be limited to the ripening stage while the Smirnoff-Wheeler pathway is operative throughout fruit development (e.g., [23]). In addition, tracer studies have suggested that the d-galacturonate pathway may contribute only moderately to fruit Asc content [32] while its contribution in other organs has not been examined. In the second alternative pathway, GDP-mannose 3′,5′-epimerase, which catalyzes conversion of GDP-d-mannose to GDP-l-galactose in the l-galactose pathway [10], also catalyzes the 5′-epimerization of GDP-d-mannose to produce GDP-l-gulose [33] (Figure 1). Conversion of GDP-l-gulose to l-gulonic acid allows Asc synthesis essentially as described in the animal pathway although evidence for this is still lacking. The presence of l-gulonic acid and l-gulono-1,4-lactone dehydrogenase activity [33,34] supports the existence of this pathway in plants. The expression of l-gulono-1,4-lactone oxidase (GulLO) from rat in lettuce and tobacco increased Asc content up to 7-fold [35] and reversed the reduction in Asc content in *Arabidopsis* mutants affected in the Smirnoff–Wheeler pathway [36] although it is not known whether l-gulono-1,4-lactone or l-galactono-1,4-lactone served as the substrate as the possibility that l-galactono-1,4-lactone served as the substrate was not examined. Although feeding with l-gulono-1,4-lactone did not increase the Asc content of tomato fruit at any developmental stage [31], its conversion to Asc has been reported for several plant species [28,37,38], supporting the presence of this pathway in plants. Expression of a foreign gene, however, can result in the ectopic expression of a pathway or the introduction of a novel pathway. Therefore, the degree to which this pathway functions in plants remains to be determined. Demonstrating that a labeled precursor directly labels Asc or that mutating a specific enzyme decreases Asc would provide more compelling evidence for such pathways.

The third alternative pathway involves d-glucuronic acid, an intermediate of the animal pathway which in plants can be generated by *myo*-inositol oxygenase (Figure 1). Support for this pathway in plants comes from the observation that overexpressing an *Arabidopsis* gene having homology to a porcine *myo*-inositol oxygenase increased Asc content [39]. As *myo*-inositol does not function as a precursor of Asc in strawberry fruit or in parsley leaves [32], this raises the question of the extent to which this pathway contributes to Asc content in plants. Nevertheless, the ability to increase Asc through the overexpression of this putative *myo*-inositol oxygenase gene may provide another strategy for increasing Asc biosynthesis.

Although multiple Asc biosynthetic pathways may exist in plants, the observation that mutants affected in the Smirnoff–Wheeler pathway result in substantial reductions in Asc content does indicate that the alternative pathways are unable to compensate for the loss in Asc biosynthetic capacity in Smirnoff–Wheeler pathway mutants. Thus, these alternative pathways may make only minor contributions to Asc biosynthesis and strategies focusing on these other pathways may be limited to increasing Asc in specific organs or at specific developmental stages.

## 3. Increasing Vitamin C Content through Improved Asc Recycling

### 3.1. Targeting MDAR Expression to Increase Ascorbic Acid

Once used in enzymatic or non-enzymatic reactions, Asc is oxidized to monodehydroascorbate (MDHA). Asc can be regenerated from MDHA through reduction by several means. If MDHA is produced in the chloroplast stroma, it can be recycled to Asc by ferredoxin (Fd), which is part of the photosynthetic electron transport chain, or by monodehydroascorbate reductase (MDAR) in the stroma [40]. Other MDAR isoforms are present in the cytosol, peroxisome, and mitochondria which reduce MDHA produced in those compartments. As no MDAR isoform is in the thylakoid lumen, MDHA cannot be reduced by MDAR or by Fd, which lies on the stromal side of the thylakoid membrane. As a result, this short-lived radical spontaneously disproportionates rapidly to Asc and DHA, particularly when the pH of the thylakoid lumen is low which occurs during the light driven transport of protons across the thylakoid membrane from the stroma into the lumen [40,41]. Under these conditions, the high pH of the stroma slows the disproportionation of MDHA and it undergoes reduction primarily through Fd or MDAR. Once photoreduced by PsaC in the PSI complex, Fd reduces MDHA directly or alternatively reduces NADP^+^ to NADPH as catalyzed by Fd-NADP^+^ reductase (FNR) which MDAR uses (or NADH instead of NADPH) to reduce MDHA to Asc [42,43]. Fd reduces MDHA at a rate that is 34-fold greater than the rate of photoreduction of NADP^+^ so that MDHA is likely reduced through Fd as part of the thylakoid scavenging system rather than by stromal MDAR when it is produced proximal to the thylakoid membrane [42]. MDAR, however, is available to reduce any stromal MDHA produced distal to the thylakoid membrane as part of the stromal scavenging system.

The multiple isoforms of MDAR are encoded by a five member nuclear gene family in *Arabidopsis* (referred to as *AtMDAR1* through *AtMDAR5*) that are targeted to the cytosol, chloroplast, mitochondria, and peroxisomes [44]. Dual targeting of MDAR to chloroplasts and mitochondria results from the use of at least two transcription start sites which produce a seven amino acid extension in the mitochondrial-targeted form of the protein [45]. The 47-kDa AtMDAR1 and 54-kDa AtMDAR4 isoforms contain a *C*-terminal sequence that targets them to the peroxisomal matrix (PTS1) and peroxisomal membrane, respectively [46]. MDAR isoforms targeted to peroxisomes, chloroplasts, or mitochondria typically function together with ascorbate peroxidase (APX) to scavenge H_2_O_2_ [47] through the transfer of electrons from two molecules of Asc to H_2_O_2_ to form water and two molecules of MDHA. Disproportionation of H_2_O_2_ is also catalyzed by catalase (CAT) when present, e.g., in the peroxisome.

Increasing Asc content by targeting MDAR expression has achieved only limited success. Expression of a cytosolic tomato MDAR from a constitutive promoter in tomato (var. Micro-Tom) resulted in a reduction in Asc in mature green tomato fruits but unaltered foliar Asc content [48] although it may improve the chilling tolerance of fruit [49]. Improved tolerance against salt and osmotic stresses was also observed following an increase in MDAR expression in tobacco [50]. Increasing expression of a tomato chloroplast-targeted MDAR in tomato increased Asc marginally (1.2-fold) but was accompanied by a decrease in DHA, resulting in an approximate doubling of the Asc redox state [51]. Similar results were obtained following the expression of an *Arabidopsis* cytosolic MDAR in tobacco [52]. The little work that has been reported to date suggests that increasing MDAR expression may achieve only minor increases in Asc content.

### 3.2. Targeting DHAR Expression to Increase Ascorbic Acid

**Figure 2:**
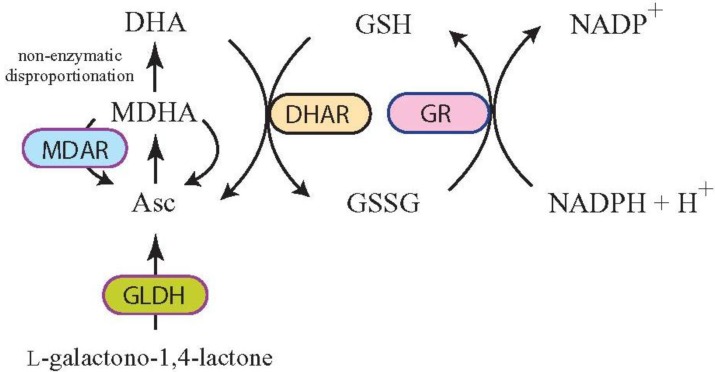
l-Ascorbic acid recycling through DHAR and MDAR. Following Asc synthesis from l-galactono-1,4-lactone by l-galactono-1,4-lactone dehydrogenase (GLDH) and oxidization to monodehydroascorbate (MDHA), monodehydroascorbate reductase (MDAR) can reduce MDHA to Asc. Alternatively, two MDHA molecules can disproportionate non-enzymatically to Asc and dehydroascorbate (DHA). Dehydroascorbate reductase (DHAR) can reduce DHA to Asc using glutathione (GSH) as the reductant. Oxidized glutathione (GSSG) is reduced by glutathione reductase (GR) to GSH using NADPH as the reductant. DHA will spontaneously hydrolyze to 2,3-diketogulonic acid if not reduced by DHAR.

If MDHA is not enzymatically reduced by Fd or MDAR, it will undergo spontaneous disproportionation to Asc and DHA, the rate of which is dependent on the pH, such as in the thylakoid lumen where Fd and MDAR are absent and the pH is low during light exposure. Disproportionation of MDHA can also occur in other cellular compartments if not enzymatically reduced. The DHA produced can be reduced to Asc by dehydroascorbate reductase (DHAR) using glutathione (GSH) as the reductant [53,54] (Figure 2). If it is not rapidly reduced, DHA undergoes irreversible hydrolysis to 2,3-diketogulonic acid and, as this is unable to be converted to Asc, it is lost to the Asc pool. Increasing the level of DHAR activity, therefore, limits DHA degradation by improving its recycling back into Asc before it is lost. As DHAR activity determines the relative levels of DHA and Asc and the enzyme is expressed in rate-limiting amounts in plants, it serves as a major regulator of the Asc redox state [5,55,56,57].

DHAR is encoded by three gene members in *Arabidopsis*: (*AtDHAR1*; At5g16710), (*AtDHAR3*; At1g75270), and (*AtDHAR5*; At1g19570) [44]. Microarray expression analysis suggests another gene, At5g36270 (*AtDHAR2*), is likely a pseudogene as it may not be expressed [44]. A fifth gene, At1g19550 (*AtDHAR4*), is smaller than other DHAR paralogs due to multiple deletions throughout the polypeptide. AtDHAR3 is likely cytoplasmic while AtDHAR5 and AtDHAR1 are targeted to the mitochondria and chloroplast, respectively [58]. No DHAR isoform is transported to the apoplast. Consequently, any Asc transported to the apoplast is quickly oxidized, disproportionates, and the resulting DHA is either degraded or transported to the cytoplasm for recycling into Asc.

Because DHAR is a major recycler of Asc, a number of studies have focused on increasing the expression of this enzyme as a means to increase Asc content in plants, which has achieved success in several species. Although ectopic expression of a human DHAR in tobacco chloroplasts failed to increase Asc despite a 2-fold increase in DHAR activity [59,60], expression of a cytosolic wheat DHAR in tobacco did increase Asc content up to 4-fold as well as the redox state (*i.e.*, an increase in the Asc to DHA ratio) with a simultaneous increase in Asc and a decrease in DHA [5]. Similar results were obtained when this cytosolic wheat DHAR was expressed in leaves and developing kernels of maize [5], demonstrating that changes in Asc can be made in photosynthetic and non-photosynthetic organs. Because Asc is transported from the cytoplasm to the apoplast and apoplastic DHA is transported back to the cytoplasm, expression of the cytosolic wheat DHAR not only increased the Asc content of the cytosol but the apoplast as well when measured from the apoplastic fluid [55], demonstrating that cytosolic DHAR regulates the symplastic and apoplastic Asc pool size and redox state. No change in Asc biosynthesis was observed following the increase in DHAR expression indicating that the synthesis of Asc and its recycling are independently controlled. The increase in DHAR expression and Asc recycling was accompanied by an increase in the GSH pool size and redox state [5]. As GSH is used by DHAR as the reductant, this suggests that the GSH pool size is affected by changes in DHAR activity. The extent to which the increase in GSH contributed to any physiological changes in these plants was not examined.

As the Asc pool size is determined by the rate of its synthesis and decay, the ability of DHAR to increase Asc content is a consequence of its recycling function that reduces DHA before it is lost through decay. As increasing DHAR expression results in improved Asc recycling and higher Asc levels, the endogenous level of DHAR is likely rate-limiting. Whether this is generally true throughout plant species is unknown. Consequently, the potential to increase Asc through increased DHAR expression will be greatest for species in which DHAR expression is rate-limiting. The strategy of increasing Asc content through increased DHAR expression, however, has been validated by subsequent studies that increased DHAR expression in the cytosol or in the chloroplast of a variety of species. Two studies that expressed a cytosolic DHAR from *Arabidopsis* in tobacco reported increases in Asc content by nearly 2-fold [52,61] whereas expression of an *Arabidopsis* cytosolic DHAR in *Arabidopsis* increased foliar Asc by 2 to 4.25-fold [62]. Expression of a rice cytosolic DHAR in *Arabidopsis* resulted in a slight increase in Asc content [63] as did expression of a rice DHAR in transformed tobacco chloroplasts [64]. Further increases in Asc content were observed when chloroplasts were transformed with glutathione reductase (GR) and DHAR [64].

Grains represent the most important food group supporting the global population either directly or indirectly as use in animal feed. Improving the nutritional value of grains offers the greatest potential for improving the diet of many and recent research has focused on engineering increasing multiple vitamins and micronutrients in grains as an efficient delivery mechanism for those whose diets are deficient in several vitamins. Although Asc content in grains is typically low, it is present during grain development but undergoes progressive oxidation during late development and is largely present as DHA by maturity [65]. The relationship between increased DHAR expression and increased Asc content in cereals was first shown in developing maize grain [5]. This was followed by a combinatorial approach to increase the levels of Asc, folate and β-carotene in maize grain using a barley d-hordein promoter to drive expression of a rice DHAR and an *E. coli* GTP cyclohydrolase (*folE*) to increase the level of Asc and folate, respectively, and a wheat LMW glutenin promoter to drive expression of maize phytoene synthase (*psy1*) and the d-hordein promoter to drive expression of *Pantoea ananatis* carotene desaturase (*crtI*) in order to increase β-carotene content [7]. These transgenes resulted in a 6-fold increase in Asc, a 2-fold increase in folate, and a 169-fold increase in β-carotene [7], demonstrating that an increase in Asc content can be combined with increases in the level of other vitamins to improve substantially the nutritional value of a fundamentally important staple. Increasing Asc content in other important, non-grain foods has been reported. Expression of a potato cytosolic DHAR from the CaMV 35S promoter increased foliar Asc content by more than 1.6-fold and in tubers by more than 1.2-fold which correlated with its expression where it is expressed higher in tubers than in leaves [66]. Expression of a chloroplast-localized potato DHAR increased foliar Asc content up to 1.5-fold but not in tubers which also correlated with its expression in leaves but a lack of expression in tubers [66]. Therefore, the strategy of increasing Asc content through increased Asc recycling through chloroplast-targeting of DHAR expression is likely to be limited to photosynthetically active tissues whereas increasing Asc content in non-photosynthetic organs will likely require expression of a cytosolic isoform of DHAR. Supporting this conclusion were results from the expression of a sesame DHAR under the control of a patatin promoter in potato. Just as the patatin promoter is active in tubers but not in leaves, expression of the sesame DHAR increased Asc 1.1 to 1.3-fold in tubers but no increase was observed in leaves. In contrast, expression of sesame DHAR under the constitutively active CaMV 35S promoter increased Asc content in leaves by 1.5-fold and 1.6-fold in tubers [67]. Overexpressing a cytosolic tomato DHAR from a constitutive promoter in tomato (var. Micro-Tom) increased Asc content in mature green and red ripe fruit by 1.6-fold in plants grown under low light [48]. In this example, however, no increase in foliar Asc was observed. The increase in Asc and GSH observed during the initial phases of embryogeny in Norway spruce following overexpression of the class I homeobox of knox 3 gene, HBK3, was attributed to increased activities of DHAR, GR, and ascorbate free radical reductase [68], suggesting that DHAR may also contribute to regulating Asc content in gymnosperm species.

## 4. Consequences of Increasing Asc Content in Plants

### 4.1. Effects on Other Antioxidants and ROS-Detoxifying Enzymes

As a major antioxidant in plants, changes in Asc content may well affect other antioxidant pools. Moreover, different approaches used for increasing Asc might be expected to affect specific antioxidants disproportionately. For example, increasing Asc by increasing biosynthetic activity would impact different antioxidant pools than would increasing Asc through improved Asc recycling which requires GSH and NADPH (or NADH) for the reduction of DHA or MDHA by DHAR or MDAR, respectively. Although most reports have observed increases in Asc content and/or in the Asc redox state following an increase in DHAR expression, the impact of this increase of DHAR activity on other antioxidants is less clear. Increases in GSH were observed in tobacco and maize expressing wheat DHAR with no change in glutathione reductase (GR), superoxide dismutase (SOD), APX, or CAT activities [5], suggesting coordinate regulation between DHAR and GSH. A similar increase in GSH content was reported for *Arabidopsis* overexpressing DHAR [62]. In contrast, expression of human DHAR in tobacco chloroplasts resulted in a reduction in GSH that was accompanied by a 1.43-fold increase in GR activity [59]. Whether this was a consequence of expression of DHAR in chloroplasts remains to be determined.

### 4.2. Increasing Ascorbic Acid Improves Tolerance to Many Environmental Stresses

Although oxygen is essential to plants, it can be highly damaging, particularly as singlet oxygen (^1^O_2_) or in its reactive forms such as the superoxide anion (O_2_^•−^), hydroxyl radical (^•^OH), or hydrogen peroxide (H_2_O_2_). ROS are detoxified through the action of antioxidants such as Asc and GSH either directly or in reactions catalyzed by SOD, APX, and CAT [69,70]. Under conditions of excess light, O_2_^•−^ is produced during photosynthesis and is converted by SOD to H_2_O_2_ which is reduced to H_2_O by APX as one means to maintain electron flow through the photosystems [71]. Abiotic stresses such as cold, drought, or high light increase ROS production by creating conditions of light stress at lower light levels. H_2_O_2_ rapidly inactivates APX if Asc is limiting [72] and inhibits CO_2_ assimilation by inhibiting several Calvin cycle enzymes [40]. ROS can invade a plant in the form of environmental pollutants, e.g., ozone [73,74], which damages cell membranes or induces programmed cell death [75,76,77]. As a defense mechanism, H_2_O_2_ produced from ozone functions as a signaling intermediate in guard cells to promote stomatal closure thus limiting ozone entry into the leaf interior [78,79].

As an antioxidant, Asc would be expected to affect tolerance to environmental stress. This was first demonstrated using *vtc* mutants of *Arabidopsis* in which their reduced Asc content correlated with a reduction in tolerance to environmental ROS. With 70%–75% less Asc, the *vtc1* mutant is hypersensitive to ozone and sulfur dioxide [13,19,80] and contains a higher oxidative load relative to wild-type plants when exposed to stress conditions such as salt despite its increased GSH content [81]. The expression level of regulators of Asc biosynthesis can also affect the degree of ozone tolerance. Knockout mutants of AMR1 (for ascorbic acid mannose pathway regulator 1) resulted in up to 3-fold greater foliar Asc content in *Arabidopsis* and increased ozone tolerance [82]. In contrast, plants with increased expression of AMR1 through activation-tagging exhibited a 60% reduction in Asc and greater ozone sensitivity [82]. As AMR1 coordinately regulates transcript expression of six Smirnoff–Wheeler pathway enzyme genes to negatively regulate Asc biosynthesis, targeting regulators of biosynthetic pathways offers yet another promising approach to alter Asc content. In a second study, overexpression of the *Arabidopsis* ethylene response factor gene AtERF98 increased Asc content up to approximately 1.6-fold which was attributed primarily to an increase in the expression of genes in the Smirnoff–Wheeler pathway [83]. As AtERF98 binds to the promoter of *VTC1*, AtERF98 likely functions as a transcriptional activator of one or more genes in the Smirnoff–Wheeler pathway [83]. Increasing AtERF98 expression resulted in enhanced salt tolerance, demonstrating that increasing Asc biosynthesis improves tolerance to this abiotic stress [83].

That the endogenous level of apoplastic Asc is important in detoxifying ozone was shown in tobacco in which the level of apoplastic Asc was specifically altered [84]. Overexpressing an apoplastic-localized cucumber ascorbate oxidase (AO), which oxidizes apoplastic Asc, increased the ozone sensitivity of transgenic tobacco, correlating with the conversion of virtually all apoplastic Asc to DHA and depriving the apoplast of its ability to detoxify ozone entering the leaf interior [84]. A decrease in the cytosolic Asc redox state was also observed which would compromise the ability of a cell to detoxify ozone entering the cytosol.

Increased sensitivity to ozone following a reduction in Asc recycling was observed following loss of cytosolic DHAR expression in the *Arabidopsis* *AtDHAR3* mutant [58]. The lower redox state but not pool size of Asc in this mutant indicates that Asc recycling is important in preventing oxidative damage. Consistent with its role in ozone tolerance, *AtDHAR3* expression is induced by ozone [58].

If decreasing Asc content reduces tolerance to environmental ROS, increasing Asc content would be predicted to have the opposite effect, a notion supported by several studies published to date. Increasing Asc content in tobacco by increasing DHAR expression increased the Asc content of the apoplast and symplast and thus increased tolerance to ozone by reducing the oxidative load of the plant (*i.e.*, a lower level of foliar and apoplastic H_2_O_2_) which was accompanied by a lower induction of antioxidant-related enzyme activities, more chlorophyll, and a higher level of photosynthetic activity following ozone exposure [56]. This increase in tolerance occurred despite the guard cells being less responsive to ozone as a consequence of their higher Asc content which reduces H_2_O_2_ levels [73,85]. Thus, increasing Asc content throughout a plant reduces guard cell responsiveness which permits more ozone to enter the leaf interior. The increased ozone tolerance can be understood, however, by the increased ability of every cell to detoxify ozone invading the leaf interior. Consistent with these findings, increasing Asc content 2-fold in tobacco through the expression of a cytosolic *Arabidopsis* DHAR enhanced its tolerance to ozone as well as drought, salt, or polyethylene glycol [61].

Conversely, a reduction in Asc recycling through the suppression of DHAR expression increased the responsiveness of guard cells to ozone thereby limiting ozone diffusion into the leaf interior [56]. At the same time, however, the decrease in DHAR activity lowered the Asc content of leaf cells and thus reduced their ability to detoxify any ozone that did invade [56]. Thus, increasing Asc content provides greater protection against environmental oxidative damage without compromising photosynthetic activity than does increasing guard cell responsiveness through decreasing Asc which reduces ozone entry but also reduces photosynthetic activity.

In addition to ozone, increasing Asc content provides greater tolerance to other environmental stresses. *Arabidopsis* with increased Asc content and redox state resulting from an increase in DHAR expression retained more Asc and chlorophyll with less membrane damage following exposure to high light and temperature or following treatment with paraquat [62]. *Arabidopsis* expressing a rice DHAR had greater tolerance to salt stress despite the small increases in DHAR activity and Asc achieved although no difference in cold tolerance was observed [63]. Although tobacco expressing a chloroplast-targeted human DHAR failed to increase Asc, it did increase the Asc redox state and the plants experienced less membrane damage following exposure to methyl viologen or H_2_O_2_ and had improved tolerance to low temperature and salt [60]. Combining expression of a chloroplast-localized DHAR with the expression of a chloroplast-localized CuZnSOD and APX increased the Asc and GSH redox states and the plants exhibited greater tolerance to paraquat and salt [86]. Greater tolerance to salt and cold was also observed in tobacco following the simultaneous expression of two pairs of chloroplast-localized enzymes, *i.e.*, an *E. coli* GR with either an *E. coli* glutathione-S-transferase (GST) or a rice DHAR, that increased Asc and GSH content and their redox states [64].

Because fewer studies on increasing Asc through MDAR expression have been reported and those that have been carried out have observed smaller increases in Asc content, much less is known about the effects of MDAR-mediated increases in Asc on plant growth and plant responses. However, the results to date suggest that increasing Asc through MDAR expression has similar effects to those following an increase in DHAR expression. The slight increase in Asc content and decrease in DHA content that resulted in an approximate doubling of the Asc redox state in tomato seedlings overexpressing a chloroplast-targeted tomato MDAR resulted in a reduced oxidative load (as measured by H_2_O_2_), lower thiobarbituric acid reactive substance (TBARS) content (a measure of membrane damage), a higher net photosynthetic rate, higher maximal photochemical efficiency of PSII and greater fresh weight when subjected to low or high temperature stress [51]. Reducing Asc and its redox state through the suppression of MDAR expression resulted in largely opposite phenotypes [51]. In agreement with these results, greater tolerance to ozone, reduced H_2_O_2_ levels, and increased photosynthetic activity were observed in tobacco expressing an *Arabidopsis* MDAR following salt stress [50].

ROS can also be generated during development. For example, H_2_O_2_ is produced in the peroxisome of oilseeds as a by-product of fatty acid β-oxidation during lipid catabolism that accompanies seedling growth [87,88]. Catalase in the peroxisomal matrix detoxifies H_2_O_2_ and a membrane-bound APX3 and MDAR4, encoded by *SUGAR-DEPENDENT2* (*SDP2*), together detoxify H_2_O_2_ using Asc [87,89,90,91]. Loss of MDAR4 expression in the *Arabidopsis* *sdp2* mutant is conditionally seedling-lethal as MDAR activity is needed to reduce leakage of H_2_O_2_ from peroxisomes that protects *SDP1*-encoded triacylglycerol (TAG) lipase activity and storage oil hydrolysis in the closely associated oil bodies during seedling growth [92]. Loss of MDAR4 activity results in inactivation of TAG lipase by H_2_O_2_ and a reduced ability to catabolize storage oil needed to support seedling growth [92]. Whether increasing Asc through increasing MDAR4 expression might improve seedling growth has not been examined. However, increasing APX3 expression increases tolerance against oxidative stress [93], suggesting an increase in Asc and the peroxisomal-associated APX3 and MDAR4 that use and recycle Asc may improve seedling tolerance against oxidative stress.

### 4.3. Increasing Ascorbic Acid Improves Tolerance to High Light

As mentioned above, in addition to environmental sources, ROS is generated during exposure to high light. Excess light energy can generate triplet state chlorophyll (^3^Chl) which transfers its energy to ground-state O_2_ to produce ^1^O_2_. Photosystem over reduction also produces ROS such as O_2_^•−^ and H_2_O_2_ [94] which can damage proteins, membranes, and pigments of photosystem I (PSI) and photosystem II (PSII), resulting in the inactivation of reaction centers as well as compromise their repair [95,96]. An increase in DHAR expression in tobacco resulted in less photoinhibition following exposure to high light that was likely due to an increase in the foliar levels of xanthophyll pigments and chlorophyll as well as in the electron transport rate (ETR) and CO_2_ assimilation, particularly at high light intensities, while ROS were reduced [97]. Thus, an increase in Asc maintains photosynthetic functioning by limiting ROS-mediated damage. Conversely, reducing Asc through suppression of DHAR results in elevated ROS and photoinhibition that is accompanied by reductions in the quantum yield of PSII and ETR [97].

### 4.4. Increasing Ascorbic Acid Decreases Tolerance to Drought Stress

While ROS are generally detrimental they also serve as important signaling cues about the external environment, e.g., the role of H_2_O_2_ in guard cells in regulating gas exchange and transpiration in response to water availability [98]. Abscisic acid (ABA) can promote H_2_O_2_ production during periods of water limitation which signals for stomatal closure [99]. Although tobacco overexpressing DHAR grew normally under well-watered conditions, the higher Asc content in guard cells not only reduced their responsiveness to ozone but also their responsiveness to the onset of water stress which normally triggers stomatal closure to prevent further water loss [55]. The reduction in responsiveness can be understood through the role of Asc as a scavenger of H_2_O_2_ and the balance between H_2_O_2_ production and Asc establishes whether H_2_O_2_ rises to a level that triggers stomatal closure. As a consequence, increasing Asc in DHAR-overexpressing tobacco maintains H_2_O_2_ at a lower level which delays stomatal closure upon onset of water stress, resulting in greater open stomatal area, increased transpiration and water loss, and ultimately decreased tolerance to water stress [55]. Reducing Asc content through suppressing DHAR expression results in an elevated accumulation of H_2_O_2_ in guard cells and a greater degree of stomatal closure even under non-stress conditions [55]. This hyperresponsiveness enables such plants to reduce transpiration during drought conditions resulting in up to 30% less water loss [55]. Thus, increasing Asc content throughout a plant confers protection against environmental ROS while reducing drought tolerance whereas reducing Asc content reduces CO_2_ assimilation under normal growth conditions as a consequence of the reduction in the open stomatal area but also reduces water loss resulting in improved drought tolerance. A strategy to increase foliar Asc content while maintaining normal levels of Asc in guard cells may improve nutritional value and tolerance to environmental ROS without increasing sensitivity to drought conditions.

### 4.5. Increasing Ascorbic Acid Prolongs Leaf Function

Increasing Asc through increasing DHAR expression resulted in higher levels of ribulose bisphosphate carboxylase/oxygenase large subunit (RbcL), chlorophyll, and CO_2_ assimilation but this had no effect on plant growth under normal conditions [57]. In contrast, reducing Asc content through reduced biosynthesis resulted in slower shoot growth, smaller leaves, and reduced shoot fresh weight and dry weight [80] while plants with lower Asc content following suppression of DHAR expression exhibited a slower rate of leaf expansion, slower shoot growth, delayed flowering time, and reduced foliar dry weight [57]. These phenotypes correlated with reduced leaf function as measured by a disproportionate loss in chlorophyll a, a reduction in RbcL, and a lower rate of CO_2_ assimilation [57]. The lower rate of CO_2_ assimilation was not due to a limitation in CO_2_ diffusion into DHAR-suppressed leaves as the sub-stomatal CO_2_ concentration was actually higher [57]. Rather, the reduced growth likely resulted from a premature loss of leaf function and early onset of senescence in mature leaves that may have reduced photosynthate available to young leaves.

### 4.6. Increasing Ascorbic Acid Can Alter Pathogen Defense Responses

The role of Asc in pathogen defense has received only limited attention. In an early study, the reduced Asc content of *Arabidopsis* *vtc1* or *vtc2* mutants resulted in reduced growth of the bacterial pathogen *Pseudomonas syringae* pv *maculicola* ES4326 and hyphal growth of the fungal pathogen *Peronospora parasitica* pv *Noco* [100]. The reduction in growth of *P. syringae* in *vtc1* plants correlated with a greater induction of the pathogenesis-related proteins PR-1 and PR-5, increased expression from some senescence-associated gene (SAG) genes and higher levels of salicylic acid. The reduced Asc content in these mutants resulted in the premature senescence of uninfected plants with an accompanying increase in salicylic acid [100]. These observations suggest that reducing Asc content predisposed *Arabidopsis* to induce defense responses faster upon pathogen attack. Whether an increase in Asc content would have had the opposite effect on these pathogens was not examined in this study. Quite different results were observed in a more recent study that also employed the same mutants. In this study, *Arabidopsis* *vtc1* and *vtc2* were more susceptible to the pathogenic ascomycete *Alternaria brassicicola* and Asc strongly inhibited growth of fungal cultures [101]. Asc levels decreased following *A. brassicicola* infection with an increase in DHA, suggesting that Asc is being consumed during infection [101]. Given the limited number of studies focusing on the relationship between Asc content and pathogen defense, it is not possible at this point to conclude how increasing Asc content will affect defense responses. These two studies do suggest, however, that changes in Asc content may affect defense responses in a very pathogen-specific manner.

### 4.7. Increasing Ascorbic Acid Induces Twinning

In addition to being an antioxidant, Asc regulates the cell cycle by promoting G_1_ to S progression of cells, e.g., in the quiescent center of onion roots [102,103,104,105,106]. Repression of l-galactono-1,4-lactone dehydrogenase (GalLDH) expression in tobacco BY-2 cell lines resulted in 30% less Asc and a reduction in the rate of cell division and growth [107]. The ability of Asc to promote cell division had dramatic consequences when its level was elevated during early embryo development. Embryo development initiates with a transverse zygotic division to produce an apical, proembryo cell and a basal cell that gives rise to the suspensor and in most species, a single embryo develops in each seed. Increasing Asc content in tobacco by increasing DHAR expression, however, resulted in monozygotic twinning and polycotyly [108]. The twin zygotes resulted from a longitudinal instead of transverse cell division and these twin zygotes developed into embryos of equal size. Direct injection of Asc into tobacco ovaries was sufficient to induce twinning but only if delivered within the first two days after pollination during which the zygote undergoes its first division. The twinning can be understood as an Asc-induced alteration in the normal transverse division of the zygote that results in a loss of the positional cues needed for the normal differentiation of the apical cell into the embryo and the basal cell into the suspensor.

Polycotyly (*i.e.*, the development of more than two cotelydons) was also induced by Asc, either following an increase in DHAR expression or when Asc was injected at the globular stage of embryo development prior to the initiation of cotyledon development [108]. As in zygotic division, an Asc-induced alteration in cell division during the specification of cotyledon-forming fields likely is responsible for the observed polycotyly. Although Asc likely affects cell division in other tissues [102,103,104,105,106], the lack of a readily observable phenotype may make the effect of increased Asc content in other aspects of plant development less apparent.

## 5. Conclusions

From its role as an antioxidant essential for photosynthesis and for detoxifying ROS from endogenous and exogenous sources, to its role in regulating cell division and flowering, to its function as a co-factor in multiple enzymatic reactions, ascorbic acid has fundamentally enabled the colonization of land by plant species. This is likely due to the challenge that the rise in atmospheric oxygen during Earth’s past presented to multicellular organisms, which required limiting the harmful consequences of increased exposure to oxygen that a land-based existence entails. Vitamin C is critical to plants as it is unlikely they could tolerate a single day of exposure to sunlight without ascorbic acid detoxifying the ROS generated by photosynthetic activity. In contrast, animals unable to synthesize ascorbic acid, such as humans, can survive the absence of the vitamin for weeks or even months before succumbing to disease and death. Despite the importance of its role in detoxifying ROS, ascorbic acid’s functions are now so integrated into plant growth and development that its importance cannot be underestimated. Because of the complexity of its many roles, any attempts to engineer changes in ascorbic acid content in plants that improves one aspect, such as nutritional content, will require close examination of how such changes impact the overall health and performance of the plant under field conditions. In addition to the engineering approaches described above, genetic diversity within plants offers another means to increase Asc content through standard breeding approaches [109], although whether changes in Asc content through these means may limit any deleterious effects on plant growth and development is unknown at this time. The most successful strategies will undoubtedly involve highly targeted approaches to alter ascorbic acid content in specific cell types or tissues to achieve a desired end while limiting possible unintended consequences in other aspects of growth, development, and responses to biotic and abiotic stresses.

## Abbreviations

ABA: abscisic acid
AO: ascorbate oxidase
APX: ascorbate peroxidase
Asc: ascorbate
CaMV: cauliflower mosaic virus
CAT: catalase
chl: chlorophyll
DHA: dehydroascorbate
DHAR: dehydroascorbate reductase
ETR: electron transport rate
Fd: ferredoxin
GalLDH: l-galactono-1,4-lactone dehydrogenase
GulLO: l-gulono-1,4-lactone oxidase
GalUR: d-galacturonic acid reductase
GR: glutathione reductase
GSH: glutathione
GST: glutathione-S-transferase
MDA: monodehydroascorbate reductase
MDAR: monodehydroascorbate reductase
NPQ: non-photochemical quenching
φPSII: quantum yield of PSII
qE: energy-dependent NPQ
qI: photoinhibition
RbcL: ribulose bisphosphate carboxylase/oxygenase large subunit
QC: quiescent center
PSI: photosystem I
PSII: photosystem II
ROS: reactive oxygen species
SOD: superoxide dismutase
TBARS: thiobarbituric acid reactive substance
VDE: violaxanthin de-epoxidase

## References

[1] Sies H., Stahl W. (1995). Vitamins E and C, β-carotene, and other carotenoids as antioxidants. Am. J. Clin. Nutr..

[2] Levine M. (1986). New concepts in the biology and biochemisty of ascorbic acid. N. Engl. J. Med..

[3] Levine M., Cantilena C.C., Dhariwal K.R. (1995). Determination of optimal vitamin C requirements in humans. Am. J. Clin. Nutr..

[4] Chatterjee I.B. (1973). Evolution and the biosynthesis of ascorbic acid. Science.

[5] Chen Z., Young T.E., Ling J., Chang S.-C., Gallie D.R. (2003). Increasing vitamin C content of plants through enhanced ascorbate recycling. Proc. Natl. Acad. Sci. USA.

[6] Hancock R.D., Viola R. (2005). Improving the nutritional value of crops through enhancement of l-ascorbic acid (vitamin C) content: Rationale and biotechnological opportunities. J. Agric. Food Chem..

[7] Naqvi S., Zhu C., Farre G., Ramessar K., Bassie L., Breitenbach J., Perez Conesa D., Ros G., Sandmann G., Capell T. (2009). Transgenic multivitamin corn through biofortification of endosperm with three vitamins representing three distinct metabolic pathways. Proc. Natl. Acad. Sci. USA.

[8] Burns J.J., Greenberg D.M. (1967). Ascorbic Acid. Metabolic Pathways.

[9] Wheeler G.L., Jones M.A., Smirnoff N. (1998). The biosynthetic pathway of vitamin C in higher plants. Nature.

[10] Wolucka B.A., Persiau G., van Doorsselaere J., Davey M.W., Demol H., Vandekerckhove J., van Montagu M., Zabeau M., Boerjan W. (2001). Partial purification and identification of GDP-mannose 3′,5′-epimerase of *Arabidopsis thaliana*, a key enzyme of the plant vitamin C pathway. Proc. Natl. Acad. Sci. USA.

[11] Siendones E., González-Reyes J.A., Santos-Ocaña Navas P., Córdoba F. (1999). Biosynthesis of ascorbic acid in kidney bean. l-Galactono-γ-lactone dehydrogenase is an intrinsic protein located at the mitochondrial inner membrane. Plant Physiol.

[12] Bartoli C.G., Pastori G.M., Foyer C.H. (2000). Ascorbate biosynthesis in mitochondria is linked to the electron transport chain between complexes III and IV. Plant Physiol..

[13] Conklin P.L., Williams E.H., Last R.L. (1996). Environmental stress sensitivity of an ascorbic acid-deficient Arabidopsis mutant. Proc. Natl. Acad. Sci. USA.

[14] Conklin P.L., Pallanca J.E., Last R.L., Smirnoff N. (1997). l-Ascorbic acid metabolism in the ascorbate-deficient Arabidopsis mutant *vtc1*. Plant Physiol..

[15] Franceschi V.R., Tarlyn N.N. (2002). l-Ascorbic acid is accumulated in source leaf phloem and transported to sink tissues in plants. Plant Physiol..

[16] Tedone L., Hancock R.D., Alberino S., Haupt S., Viola R. (2004). Long-distance transport of l-ascorbic acid in potato. BMC Plant Biol..

[17] Conklin P.L., Saracco S.A., Norrism S.R., Last R.L. (2000). Identification of ascorbic acid-deficient *Arabidopsis thaliana* mutants. Genetics.

[18] Dowdle J., Ishikawa T., Gatzek S., Rolinski S., Smirnoff N. (2007). Two genes in *Arabidopsis thaliana* encoding GDP-l-galactose phosphorylase are required for ascorbate biosynthesis and seedling viability. Plant J..

[19] Conklin P.L., Norris S.R., Wheeler G.L., Williams E.H., Smirnoff N., Last R.L. (1999). Genetic evidence for the role of GDP-mannose in plant ascorbic acid (vitamin C) biosynthesis. Proc. Natl. Acad. Sci. USA.

[20] Conklin P.L., Gatzek S., Wheeler G.L, Dowdle J., Raymond M.J., Rolinski S., Isupov M., Littlechild J.A., Smirnoff N. (2006). *Arabidopsis thaliana VTC4* encodes l-galactose-1-P phosphatase, a plant ascorbic acid biosynthetic enzyme. J. Biol. Chem..

[21] Laing W.A., Wright M.A., Cooney J., Bulley S.M. (2007). The missing step of the l-galactose pathway of ascorbate biosynthesis in plants, an l-galactose guanyltransferase, increases leaf ascorbate content. Proc. Natl. Acad. Sci. USA.

[22] Bulley S.M., Rassam M., Hoser D., Otto W., Schünemann N., Wright M., MacRae E., Gleave A., Laing W. (2009). Gene expression studies in kiwifruit and gene over-expression in Arabidopsis indicates that GDP-l-galactose guanyltransferase is a major control point of vitamin C biosynthesis. J. Exp. Bot..

[23] Bulley S., Wright M., Rommens C., Yan H., Rassam M., Lin-Wang K., Andre C., Brewster D., Karunairetnam S., Allan A.C. (2012). Enhancing ascorbate in fruits and tubers through over-expression of the l-galactose pathway gene GDP-l-galactose phosphorylase. Plant Biotechnol. J..

[24] Gatzek S., Wheeler G.L., Smirnoff N. (2002). Antisense suppression of l-galactose dehydrogenase in *Arabidopsis thaliana* provides evidence for its role in ascorbate synthesis and reveals light modulated l-galactose synthesis. Plant J..

[25] Zhou Y., Tao Q.C., Wang Z.N., Fan R., Li Y., Sun X.F., Tang K.X. (2012). Engineering ascorbic acid biosynthetic pathway in *Arabidopsis* leaves by single and double gene transformation. Biol. Plant..

[26] Valpuesta V., Botella M.A. (2004). Biosynthesis of l-ascorbic acid in plants: New pathways for an old antioxidant. Trends Plant Sci..

[27] Loewus F.A., Kelly S. (1961). The metabolism of d-galacturonic acid and its methyl ester in the detached ripening strawberry. Arch. Biochem. Biophys..

[28] Isherwood F.A., Chen Y.T., Mapson L.W. (1954). Synthesis of l-ascorbic acid in plants and animals. Biochem. J..

[29] Davey M.W., Gilot C., Persiau G., Østergaard J., Han Y., Bauw G.C., van Montagu M.C. (1999). Ascorbate biosynthesis in *Arabidopsis* cell suspension culture. Plant Physiol..

[30] Agius F., Gonzalez-Lamothe R., Caballero J.L., Munoz-Blanco J., Botella M.A., Valpuesta V. (2003). Engineering increased vitamin C levels in plants by overexpression of a d-galacturonic acid reductase. Nat. Biotechnol..

[31] Badejo A.A., Wada K., Gao Y., Maruta T., Sawa Y., Shigeoka S., Ishikawa T. (2012). Translocation and the alternative d-galacturonate pathway contribute to increasing the ascorbate level in ripening tomato fruits together with the d-mannose/l-galactose pathway. J. Exp. Bot..

[32] Loewus F.A. (1963). Tracer studies on ascorbic acid formation in plants. Phytochemistry.

[33] Wolucka B.A., van Montagu M. (2003). GDP-mannose 3′,5′-epimerase forms GDP-l-gulose, a putative intermediate for the de novo biosynthesis of vitamin C in plants. J. Biol. Chem..

[34] Wagner C., Sefkow M., Kopka J. (2003). Construction and application of a mass spectral and retention time index database generated from plant GC/EI-TOF-MS metabolite profile. Phytochemistry.

[35] Jain A.K., Nessler C.L. (2000). Metabolic engineering of an alternative pathway for ascorbic acid biosynthesis in plants. Mol. Breed..

[36] Radzio J.A., Lorence A., Chevone B.I., Nessler C.L. (2004). l-Gulono-1,4-lactone oxidase expression rescues vitamin C-deficient *Arabidopsis* (*vtc*) mutants. Plant Mol. Biol..

[37] Baig M.M., Kelly S., Loewus F. (1970). l-Ascorbic acid biosynthesis in higher plants from l-gulono-1,4-lactone and l-galactono-1,4-lactone. Plant Physiol..

[38] Oba K., Fukui M., Imai Y., Iriyama S., Nogami K. (1994). l-Galactono-γ-lactone dehydrogenase: Partial characterization, induction of activity and role in the synthesis of ascorbic acid in wounded white potato tuber tissue. Plant Cell Physiol..

[39] Lorence A., Chevone B.I., Mendes P., Nessler C.L. (2004). *myo*-Inositol oxygenase offers a possible entry point into plant ascorbate biosynthesis. Plant Physiol..

[40] Asada K. (1999). The water-water cycle in chloroplasts: Scavenging of active oxygens and dissipation of excess photons. Annu. Rev. Plant Physiol. Plant Mol. Biol..

[41] Mano J., Hideg E., Asada K. (2004). Ascorbate in thylakoid lumen functions as an alternative electron donor to photosystem II and photosystem I. Arch. Biochem. Biophys..

[42] Miyake C., Asada K. (1994). Ferredoxin dependent photoreduction of monodehydroascorbate radical in spinach thylakoids. Plant Cell Physiol..

[43] Sano S., Miyake C., Mikami B., Asada K. (1995). Molecular characterization of monodehydroascorbate radical reductase from cucumber overproduced in *Escherichia coli*. J. Biol. Chem..

[44] Mittler R., Vanderauwera S., Gollery M., van Breusegem F. (2004). Reactive oxygen gene network of plants. Trends Plant Sci..

[45] Obara K., Sumi K., Fukuda H. (2002). The use of multiple transcription starts causes the dual targeting of *Arabidopsis* putative monodehydroascorbate reductase to both mitochondria and chloroplasts. Plant Cell Physiol..

[46] Lisenbee C.S., Lingard M.J., Trelease R.N. (2005). Arabidopsis peroxisomes possess functionally redundant membrane and matrix isoforms of monodehydroascorbate reductase. Plant J..

[47] Jimenez A., Hernandez J.A., del Reo L.A., Sevilla F. (1997). Evidence for the presence of the ascorbate-glutathione cycle in mitochondria and peroxisomes of pea leaves. Plant Physiol..

[48] Haroldsen V.M., Chi-Ham C.L., Kulkarni S., Lorence A., Bennett A.B. (2011). Constitutively expressed DHAR and MDHAR influence fruit, but not foliar ascorbate levels in tomato. Plant Physiol. Biochem..

[49] Stevens R., Page D., Gouble B., Garchery C., Zamir D., Causse M. (2008). Tomato fruit ascorbic acid content is linked with monodehydroascorbate reductase activity and tolerance to chilling stress. Plant Cell Environ..

[50] Eltayeb A.E., Kawano N., Badawi G.H., Kaminaka H., Sanekata T., Shibahara T., Inanaga S., Tanaka K. (2007). Overexpression of monodehydroascorbate reductase in transgenic tobacco confers enhanced tolerance to ozone, salt and polyethylene glycol stresses. Planta.

[51] Li F., Wu Q.Y., Sun Y.L., Wang L.Y., Yang X.H., Meng Q.W. (2010). Overexpression of chloroplastic monodehydroascorbate reductase enhanced tolerance to temperature and methyl viologen-mediated oxidative stresses. Physiol. Plant.

[52] Yin L., Wang S., Eltayeb A.E., Uddin M.I., Yamamoto Y., Tsuji W., Takeuchi Y., Tanaka K. (2010). Overexpression of dehydroascorbate reductase, but not monodehydroascorbate reductase, confers tolerance to aluminum stress in transgenic tobacco. Planta.

[53] Smirnoff N., Conklin P.L., Loewus F.A. (2001). Biosynthesis of ascorbic acid in plants: A Renaissance. Annu. Rev. Plant Physiol. Plant Mol. Biol..

[54] Noctor G., Foyer C.H. (1998). Ascorbate and glutathione: Keeping active oxygen under control. Ann. Rev. Plant Physiol. Plant Mol. Biol..

[55] Chen Z., Gallie D.R. (2004). The ascorbic acid redox state controls guard cell signaling and stomatal movement. Plant Cell.

[56] Chen Z., Gallie D.R. (2005). Increasing tolerance to ozone by elevating foliar ascorbic acid confers greater protection against ozone than increasing avoidance. Plant Physiol..

[57] Chen Z., Gallie D.R. (2006). Dehydroascorbate reductase affects leaf growth, development, and function. Plant Physiol..

[58] Yoshida S., Tamaoki M., Shikano T., Nakajima N., Ogawa D., Ioki M., Aono M., Kubo A., Kamada H., Inoue Y. (2006). Cytosolic dehydroascorbate reductase is important for ozone tolerance in *Arabidopsis thaliana*. Plant Cell Physiol..

[59] Kwon S.Y., Ahn Y.O., Lee H.S., Kwak S.S. (2001). Biochemical characterization of transgenic tobacco plants expressing a human dehydroascorbate reductase gene. J. Biochem. Mol. Biol..

[60] Kwon S.Y., Choi S.M., Ahn Y.O., Lee H.S., Lee H.B., Park Y.M., Kwak S.S. (2003). Enhanced stress-tolerance of transgenic tobacco plants expressing a human dehydroascorbate reductase gene. J. Plant Physiol..

[61] Eltayeb A.E., Kawano N., Badawi G.H., Kaminaka H., Sanekata T., Morishima I., Shibahara T., Inanaga S., Tanaka K. (2006). Enhanced tolerance to ozone and drought stresses in transgenic tobacco overexpressing dehydroascorbate reductase in cytosol. Physiol. Plant.

[62] Wang Z., Xiao Y., Chen W., Tang K., Zhang L. (2010). Increased vitamin C content accompanied by an enhanced recycling pathway confers oxidative stress tolerance in *Arabidopsis*. J. Integr. Plant Biol..

[63] Ushimaru T., Nakagawa T., Fujioka Y., Daicho K., Naito M., Yamauchi Y., Nonaka H., Amako K., Yamawaki K., Murata N. (2006). Transgenic *Arabidopsis* plants expressing the rice dehydroascorbate reductase gene are resistant to salt stress. J. Plant Physiol..

[64] Le Martret B., Poage M., Shiel K., Nugent G.D., Dix P.J. (2011). Tobacco chloroplast transformants expressing genes encoding dehydroascorbate reductase, glutathione reductase, and glutathione-S-transferase, exhibit altered anti-oxidant metabolism and improved abiotic stress tolerance. Plant Biotechnol. J..

[65] Arrigoni O., De Gara L., Tommasi F., Liso R. (1992). Changes in the ascorbate system during seed development of *Vicia faba* L. Plant Physiol..

[66] Qin A., Shi Q., Yu X. (2011). Ascorbic acid contents in transgenic potato plants overexpressing two dehydroascorbate reductase genes. Mol. Biol. Rep..

[67] Goo Y.M., Chun H., Kim T.W., Lee C.H., Ahn M.J., Bae S.C., Cho K.J., Chun J.A., Chung C.H., Lee S.W. (2008). Expressional characterization of dehydroascorbate reductase cDNA in transgenic potato plants. J. Plant Biol..

[68] Belmonte M.F., Stasolla C. (2009). Altered HBK3 expression affects glutathione and ascorbate metabolism during the early phases of Norway spruce (*Picea abies*) somatic embryogenesis. Plant Physiol. Biochem..

[69] Halliwell B., Gutteridge J.M.C. (2000). Free Radicals in Biology and Medicine.

[70] Foyer C.H., Scandalios J.G. (1997). Oxygen Metabolism and Electron Transport in Photosynthesis. Oxidative Stress and the Molecular Biology of Antioxidant Defenses.

[71] Asada K. (2000). The water-water cycle as alternative photon and electron sinks. Philos. Trans. R. Soc. Lond. B.

[72] Nakano Y., Asada K. (1980). Spinach chloroplasts scavenge hydrogen peroxide on illumination. Plant Cell Physiol..

[73] Mudd J.B., Yunus M., Iqba M. (1997). Biochemical Basis for the Toxicity of Ozone. Plant Response to Air Pollution.

[74] Schraudner M., Moeder W., Wiese C., van Camp W., Inze D., Langebartels C., Sandermann H. (1998). Ozone-induced oxidative burst in the ozone biomonitor plant, tobacco Bel W3. Plant J..

[75] Rao M.V., Koch J.R., Davis K.R. (2000). Ozone: A tool for probing programmed cell death in plants. Plant Mol. Biol..

[76] Koch J.R., Creelman R.A., Eshita S.M., Seskar M., Mullet J.E., Davis K.R. (2000). Ozone sensitivity in hybrid poplar correlates with insensitivity to both salicylic acid and jasmonic acid. The role of programmed cell death in lesion formation. Plant Physiol..

[77] Pasqualini S., Piccioni C., Reale L., Ederli L., Della Torre G., Ferranti F. (2003). Ozone-induced cell death in tobacco cultivar bel w3 plants. The role of programmed cell death in lesion formation. Plant Physiol..

[78] Pei Z.M., Murata Y., Benning G., Thomine S., Klusener B., Allen G.J., Grill E., Schroeder J.I. (2000). Calcium channels activated by hydrogen peroxide mediate abscisic acid signaling in guard cells. Nature.

[79] Zhang X., Zhang L., Dong F., Gao J., Galbraith D.W., Song C.P. (2001). Hydrogen peroxide is involved in abscisic acid-induced stomatal closure in *vicia faba*. Plant Physiol..

[80] Veljovic-Jovanovic S.D., Pignocchi C., Noctor G., Foyer C.H. (2001). Low ascorbic acid in the *vtc-1* mutant of Arabidopsis is associated with decreased growth and intracellular redistribution of the antioxidant system. Plant Physiol..

[81] Huang C., He W., Guo J., Chang X., Su P., Zhang L. (2005). Increased sensitivity to salt stress in an ascorbate-deficient *Arabidopsis* mutant. J. Exp. Bot..

[82] Zhang W., Lorence A., Gruszewski H.A., Chevone B.I., Nessler C.L. (2009). AMR1, an Arabidopsis gene that coordinately and negatively regulates the mannose/l-galactose ascorbic acid biosynthetic pathway. Plant Physiol..

[83] Zhang Z., Wang J., Zhang R., Huang R. (2012). The ethylene response factor Aterf98 enhances tolerance to salt through the transcriptional activation of ascorbic acid synthesis in Arabidopsis. Plant J..

[84] Sanmartin M., Drogoudi P.A., Lyons T., Pateraki I., Barnes J., Kanellis A.K. (2003). Over-expression of ascorbate oxidase in the apoplast of transgenic tobacco results in altered ascorbate and glutathione redox states and increased sensitivity to ozone. Planta.

[85] Grimes H.D., Perkins K.K., Boss W.F. (1983). Ozone degrades into hydroxyl radical under physiological conditions: A spin trapping study. Plant Physiol..

[86] Lee Y.P., Kim S.H., Bang J.W., Lee H.S., Kwak S.S., Kwon S.Y. (2007). Enhanced tolerance to oxidative stress in transgenic tobacco plants expressing three antioxidant enzymes in chloroplasts. Plant Cell Rep..

[87] Mullen R.T., Trelease R.N. (1996). Biogenesis and membrane properties of peroxisomes: Does the boundary membrane serve and protect?. Trends Plant Sci..

[88] Graham I.A., Eastmond P.J. (2002). Pathways of straight and branched chain fatty acid catabolism in higher plants. Prog. Lipid Res..

[89] Yamaguchi K., Mori H., Nishimura M. (1995). A novel isoenzyme of ascorbate peroxidase localized on glyoxysomal and leaf peroxisomal membranes in pumpkin. Plant Cell Physiol.

[90] Bunkelmann J.R., Trelease R.N. (1996). Ascorbate peroxidase: A promenent membrane protein in oilseed glyoxysomes. Plant Physiol.

[91] Karyotou K., Donaldson R.P. (2005). Ascorbate peroxidase, a scavenger of hydrogen peroxide in glyoxysomal membranes. Arch. Biochem. Biophys..

[92] Eastmond P.J. (2007). MONODEHYROASCORBATE REDUCTASE4 is required for seed storage oil hydrolysis and postgerminative growth in *Arabidopsis*. Plant Cell.

[93] Wang J., Zhang H., Allen R.D. (1999). Overexpression of an Arabidopsis peroxisomal ascorbate peroxidase gene in tobacco increases protection against oxidative stress. Plant Cell Physiol..

[94] Asada K., Takahashi M., Kyle D.J., Osmond C.B., Arntzen C.J. (1987). Production and Scavenging of Active Oxygen in Photosynthesis. Photoinhibition.

[95] Aro E.M., Virgin I., Andersson B. (1993). Photoinhibition of photosystem II. Inactivation, protein damage and turnover. Biochim. Biophys. Acta.

[96] Nishiyama Y., Allakhverdiev S.I., Murata N. (2006). A new paradigm for the action of reactive oxygen species in the photoinhibition of photosystem II. Biochim. Biophys. Acta.

[97] Chen Z., Gallie D.R. (2008). Dehydroascorbate reductase affects non-photochemical quenching and photosynthetic performance. J. Biol. Chem..

[98] Assmann S.M., Wang X.Q. (2001). From milliseconds to millions of years: Guard cells and environmental responses. Curr. Opin. Plant Biol..

[99] Schroeder J.I., Allen G.J., Hugouvieux V., Kwak J.M., Waner D. (2001). Guard cell signal transduction. Annu. Rev. Plant Physiol. Plant Mol. Biol..

[100] Barth C., Moeder W., Klessig D.F., Conklin P.L. (2004). The timing of senescence and response to pathogens is altered in the ascorbate-deficient Arabidopsis mutant *vitamin c-1*. Plant Physiol.

[101] Botanga C.J., Bethke G., Chen Z., Gallie D.R., Fiehn O., Glazebrook J. (2012). Metabolite profiling of *Arabidopsis* inoculated with *Alternaria brassicicola* reveals that ascorbate reduces disease severity. Mol. Plant Microbe Interact..

[102] Liso R., Calabrese G., Bitonti M.B., Arrigoni O. (1984). Relationship between ascorbic acid and cell division. Exp. Cell Res..

[103] Arrigoni O., Bitonti M.B., Cozza R., Innocenti A.M., Liso R., Veltri R. (1989). Ascorbic acid effect on pericycle cell line in *Allium cepa* root. Caryologia.

[104] Innocenti A.M., Bitonti M.B., Arrigoni O., Liso R. (1990). The size of quiescent centre in roots of *Allium Cepa* L. grown with ascorbic acid. New Phytol..

[105] Arrigoni O. (1994). Ascorbate system in plant development. J. Bioenerg. Biomembr.

[106] Citterio S., Sgorbati S., Scippa S., Sparvoli E. (1994). Ascorbic acid effect on the onset of cell proliferation in pea root. Physiol. Plant.

[107] Tabata K., Oba K., Suzuki K., Esaka M. (2001). Generation and properties of ascorbic acid-deficient transgenic tobacco cells expressing antisense RNA for l-galactono-1,4-lactone dehydrogenase. Plant J..

[108] Chen Z., Gallie D.R. (2012). Induction of monozygotic twinning by ascorbic acid in tobacco. PLoS One.

[109] Gest N., Gautier H., Stevens R. (2013). Ascorbate as seen through plant evolution: The rise of a successful molecule?. J. Exp. Bot..

